# Supplementary material for: Repeated Social Defeat Stress Induces HMGB1 Nuclear Export in Prefrontal Neurons, Leading to Social Avoidance in Mice
Source: Cells. 2023 Jul 5;12(13):1789. doi: 10.3390/cells12131789 (PMC10340449; doi:10.3390/cells12131789)
Supplement: Supplementary file 1 [file cells-12-01789-s001.zip › cells-2493295-supplementary.pdf]

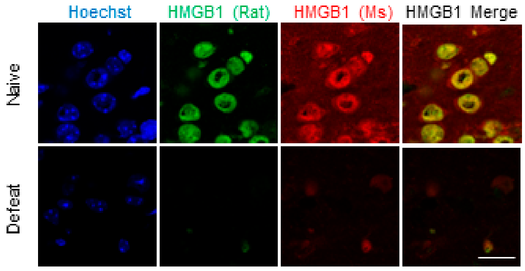

Figure S1. The specificity of anti-HMGB1 antibodies. HMGB1 immunostaining with two independent antibodies of rat and mouse origin (green and red, respectively) showed the signals colocalized in the nuclei in the mPFC of Naïve and Defeat mice at 90 min after the last session of cage transfer or social defeat stress, respectively. Nuclei were counterstained with Hoechst (blue). Scale bar, 20 μm.

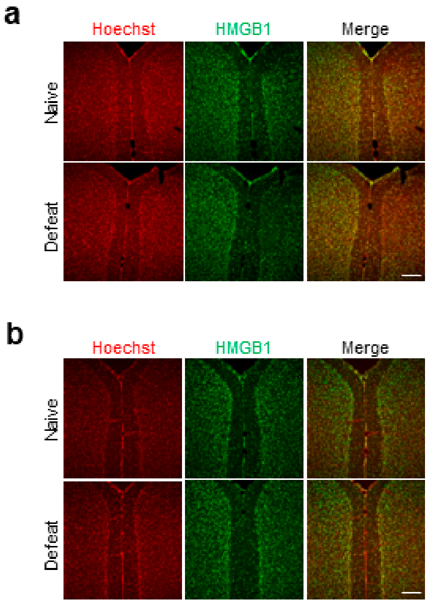

Figure S2. The time course of repeated social defeat stress-induced HMGB1 nuclear export in the mPFC. Representative images of HMGB1 immunostaining in the mPFC of Naïve and Defeat mice immediately (a) and at 24 h (b) after the last session of cage transfer or social defeat stress, respectively. HMGB1 signals are shown in green. Nuclei were counterstained with Hoechst (red). Scale bar, 100 μm.

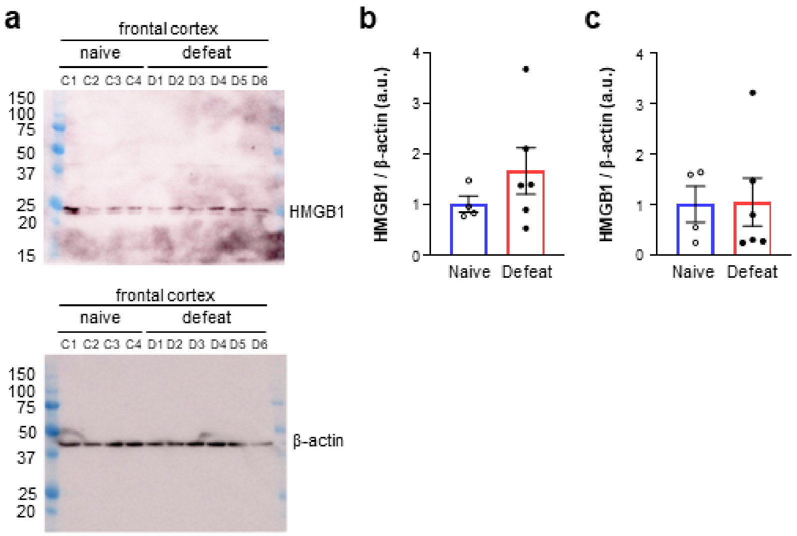

Figure S3. Western blotting of HMGB1 in mPFC whole tissue lysates shows that there is no difference in its signal intensity between the Naïve and Defeat groups. (a) Images of HMGB1 and β-actin immunoblotting of the mPFC lysates of wild-type mice. (b) Quantitative analysis of HMGB1 intensity shown in (a). Its intensity was normalized with β-actin. (c) The same samples were analyzed with the Protein Simple Abby. Data are shown as mean ± SEM.

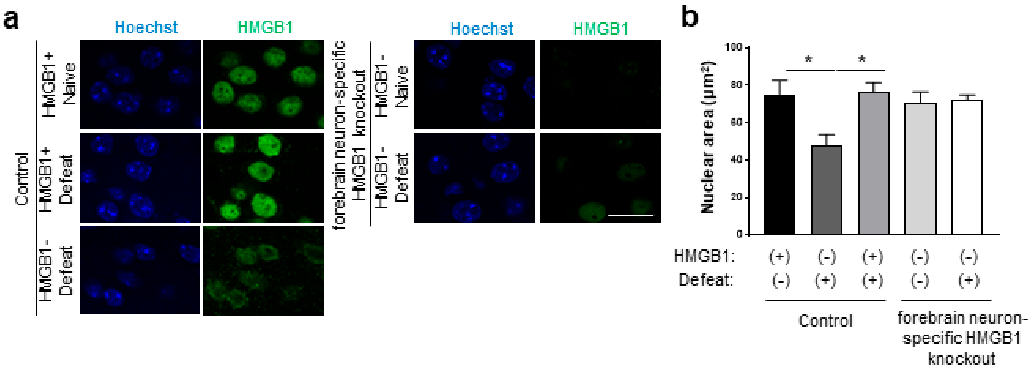

Figure S4. Repeated social defeat stress induces nuclear shrinkage of mPFC neurons in an HMGB1-dependent manner. (a) Representative images of HMGB1 immunostaining in the mPFC of control and forebrain neuron-specific HMGB1 knockout mice (HMGB1fl/fl and αCaMKII-CreERT/+; HMGB1fl/fl, respectively). For control mice in the Defeat group, both HMGB1-affected subregions and their surrounding are shown. Scale bar, 20μm. (b) Quantitative analysis of the area of nuclei in the mPFC for the mice shown in (a). Data are shown as mean ± SEM. * $P < 0.05$ for Tukey's multiple comparison test.

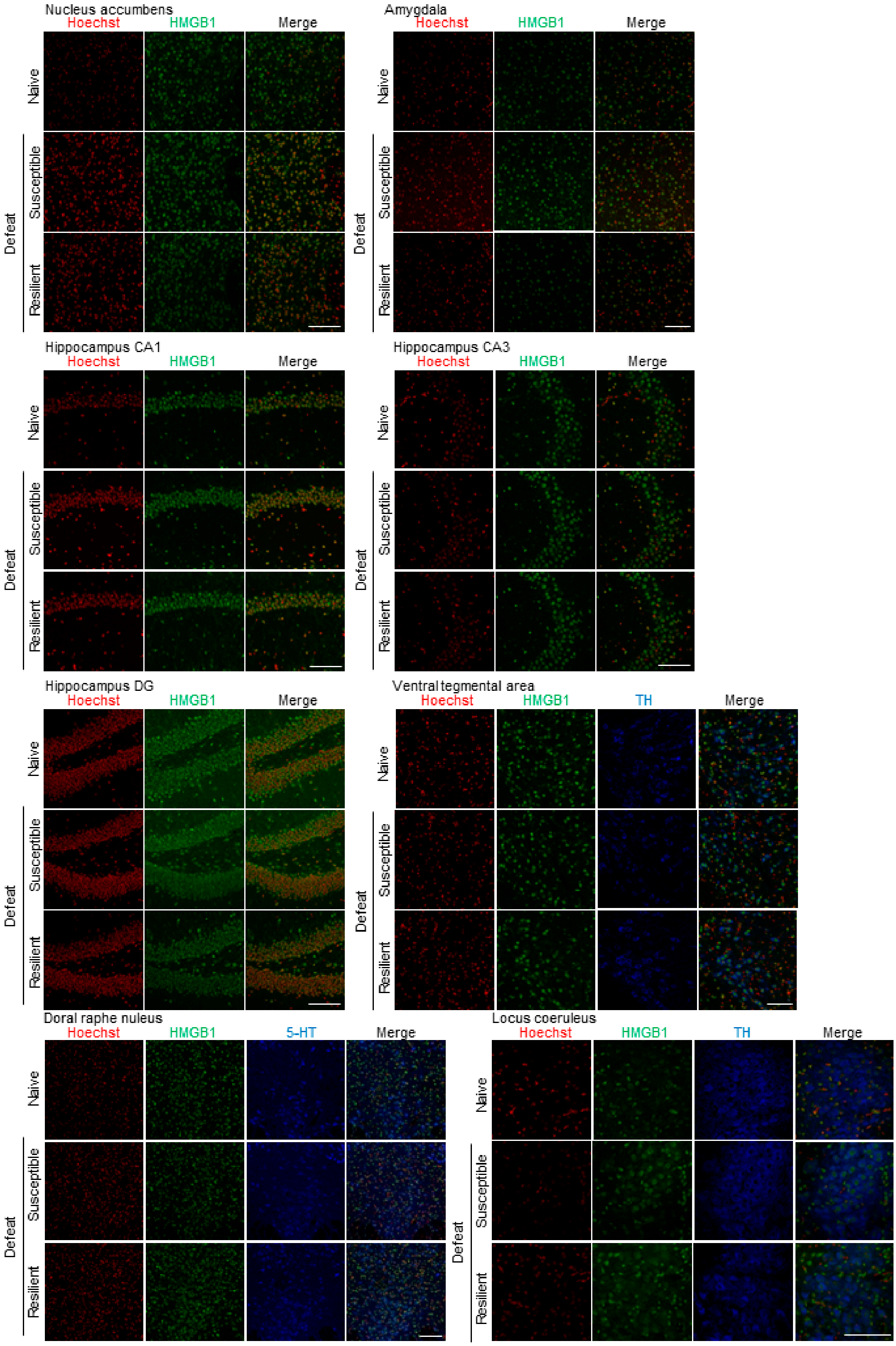

Nucleus accumbens
Hoechst
HMGB1
Merge
Naive
Susceptible
Resilient
Defeat
Amygdala
Hoechst
HMGB1
Merge
Naive
Susceptible
Resilient
Defeat
Hippocampus CA1
Hoechst
HMGB1
Merge
Naive
Susceptible
Resilient
Defeat
Hippocampus CA3
Hoechst
HMGB1
Merge
Naive
Susceptible
Resilient
Defeat
Hippocampus DG
Hoechst
HMGB1
Merge
Naive
Susceptible
Resilient
Defeat
Ventral tegmental area
Hoechst
HMGB1
TH
Merge
Naive
Susceptible
Resilient
Defeat
Doral raphe nuleus
Hoechst
HMGB1
5-HT
Merge
Naive
Susceptible
Resilient
Defeat
Locus coeruleus
Hoechst
HMGB1
TH
Merge
Naive
Susceptible
Resilient
Defeat

Figure S5a.

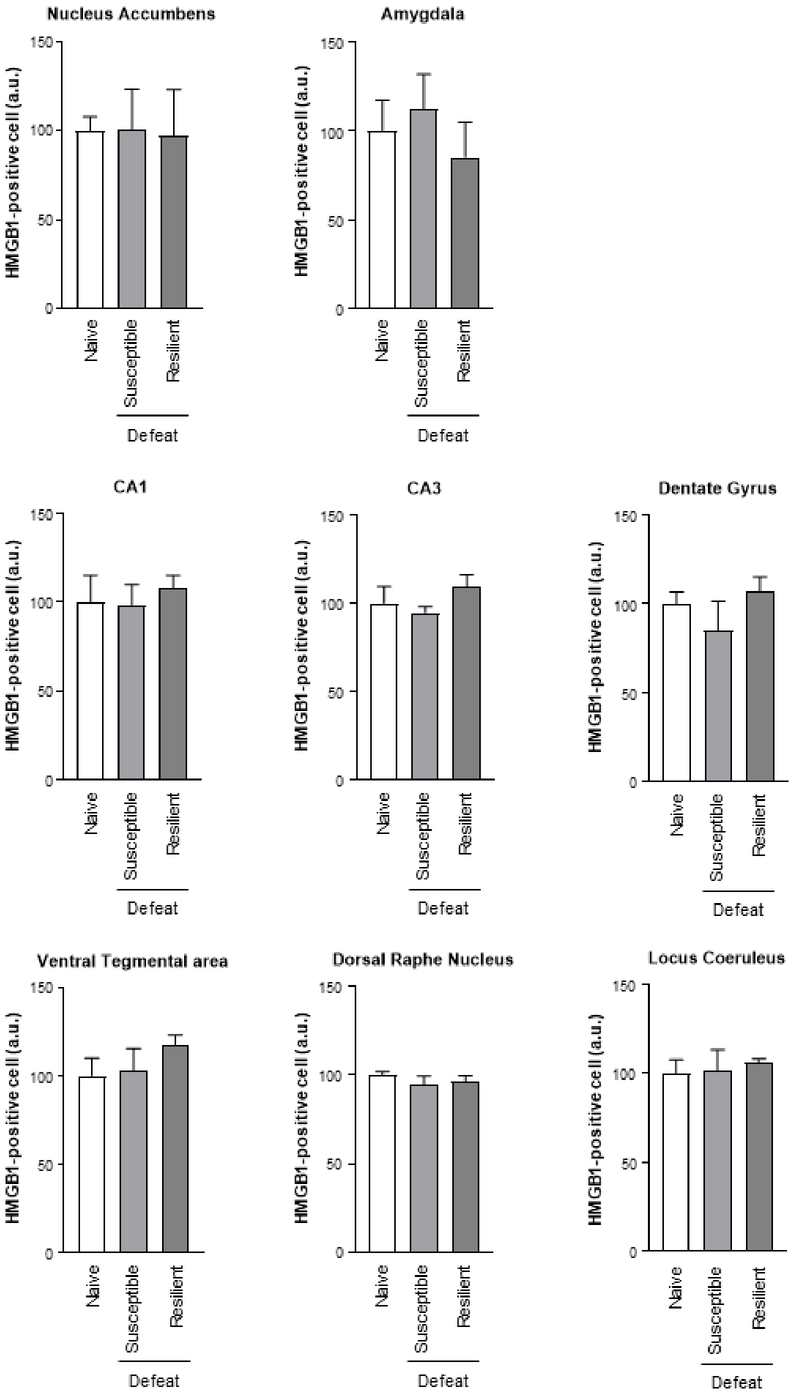

Figure S5b. Repeated social defeat stress-induced HMGB1 nuclear export is undetectable in the nucleus accumbens, ventral tegmental, amygdala, ventral hippocampus, dorsal raphe nucleus, and locus coeruleus. (a) The behavioral experiment and HMGB1 immunostaining were performed as described in the legend of Figure 5. 5-hydroxytryptamine (5-HT) and tyrosine hydroxylase (TH) were co-stained for brain regions containing monoaminergic neurons (blue). (b) The number of HMGB1-positive cells in the brain regions shown in (a) in the Naïve, susceptible, and resilient mice. The values were normalized to the average of the Naïve mice in each batch of experiments.
